# Groundwater Solute-Induced Desorption of Perfluoroalkyl Substances (PFAS) from Colloidal Activated Carbon (CAC)

**DOI:** 10.1021/acs.est.6c00984

**Published:** 2026-06-01

**Authors:** Rachel A. Molé, Adriana C Velosa, Xitong Liu, Guangbin Li, Dimin Fan, Anthony Danko, Gregory V. Lowry

**Affiliations:** † Department of Civil and Environmental Engineering, 6612 Carnegie Mellon University, Pittsburgh, Pennsylvania 15213, United States; ‡ Department of Civil and Environmental Engineering, The George Washington University, Washington, District of Columbia 20052, United States; § Department of Civil and Environmental Engineering, University of Maryland, College Park, Maryland 20742, United States; ∥ Geosyntec Consultants, Inc, 10211 Wincopin Cir Floor 4, Columbia, Maryland 21044, United States; ⊥ Naval Facilities Engineering Systems Command, Engineering and Expeditionary Warfare Center, Port Hueneme, California 93043, United States

**Keywords:** in situ PFAS remediation, carbon barrier performance, diesel range organics, PFAS release, perfluoroalkyl sulfonamide, AFFF, colloidal activated carbon (CAC)

## Abstract

In situ colloidal activated carbon (CAC) barriers limit the migration of per- and polyfluorinated alkyl substances (PFAS) in groundwater. While the influence of site-specific conditions on equilibrium adsorption has been studied, the kinetics and mechanisms of desorption remain unreported. Here, we quantify the extent of PFAS desorption from CAC using preloaded flow-through columns and explore mechanisms of release. PFAS release after 1600 pore volumes under baseline conditions (1 mM NaHCO_3_, pH = 7.5) was controlled by chain length and headgroup and varied from <1% for perfluorooctanoic acid (PFOA) to 83% for perfluoropentanoic acid (PFPeA). Release of perfluorobutane sulfonamide (FBSA) (30%) was lower than that of perfluorobutanesulfonic acid (PFBS) (46%), which was attributed to its near-neutral pK_a_ and a fraction of uncharged species with greater sorption stability. Low-molecular-weight (MW) dissolved organic matter (DOM) displaced more PFAS than high-MW DOM. Diesel range organics (DRO 1400 μg/L) led to ∼70% of the total PFOA mass recovered in effluent, which was significant. Elevated ionic strength (100 mM) did not greatly impact the total mass desorbed but did decrease the rate of PFAS mass release. Results demonstrate that previously sequestered PFAS can be released following exposure to DOM and DRO, and that nonequilibrium mass transfer rate-limited processes could impact barrier performance at sites where groundwater retention times in the barrier are below what is required for equilibrium.

## Graphical abstract

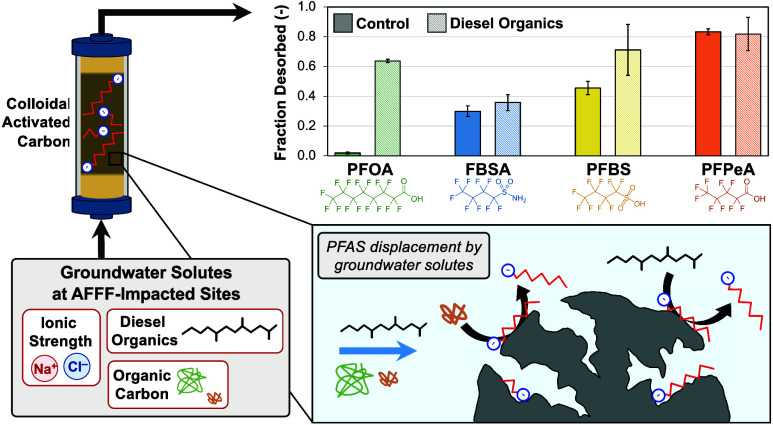

## 1 Introduction

Per- and polyfluorinated alkyl substances (PFAS) are a class of recalcitrant and anthropogenic compounds that have been identified as chemicals of concern by state and federal regulatory agencies.^1,2^ Use of aqueous film-forming foams (AFFF) for firefighting activities has led to a broad range of PFAS with different structures and chain lengths being detected in groundwater at many military and airport sites.^3,4^ In situ colloidal activated carbon (CAC) barriers are a rapidly growing approach to manage PFAS migration in groundwater. CAC particles between 0.5 and 2 μm can be placed within a PFAS source area or downgradient of a plume to limit mass flux to downgradient receptors.^5–12^ CAC barriers are intended to remain effective for years to decades. However, uncertainty around long-term barrier performance remains a primary question surrounding the use and adaptation of in situ CAC barriers, particularly regarding the potential for PFAS displacement and release under changing groundwater conditions.^10,11,13,14^

The range of PFAS structures and changes in groundwater chemistry over time may affect long-term CAC barrier performance. Many AFFF-impacted sites are also located in coastal regions where tidal pumping creates large fluctuations in ionic strength (IS) and concentrations of dissolved organic matter (DOM).^15^ Changes in water chemistry will impact equilibrium PFAS adsorption to different degrees depending on PFAS structure.^11,13^ In batch adsorption experiments, elevated IS (100 mM, NaCl) significantly decreased short-chain perfluoroalkyl acid (PFAA) adsorption to CAC due to competitive effects with inorganic anions, but had minimal impact on long-chain PFAA.^11,13,16^ DOM concentrations in coastal waters can also fluctuate over time and decrease PFAS adsorption to CAC.^13,17–19^ DOM with varying molecular weights (MW) has also been shown to impact PFAS adsorption differently, with smaller-MW DOM impacting equilibrium PFAS adsorption more than larger-MW fractions.^11,20^

Co-occurring compounds present at AFFF-impacted sites can potentially affect the long-term performance of CAC barriers. Due to historical fire-training exercises and repeated releases, AFFF-impacted sites can contain mixtures of PFAS, fuel-related compounds including benzene, toluene, ethylbenzene, xylenes (BTEX), diesel range organics (DRO), and chlorinated solvents used as burning agents.^6,21,22^ However, remediation efforts at AFFF-impacted sites can reduce the concentration of co-occurring compounds like hydrocarbons and DRO in comparison to PFAS, which are not removed by traditional biological and chemical approaches. Carey et al. present a database of 17 sites impacted by AFFF where CAC barriers have been installed and found that high hydrocarbon concentrations (>1,000 μg/L) were observed at three sites and the median total co-occurring compounds concentration was 0.21 μg/L.^6^ While these concentrations did not impact the initial success of the referenced CAC barriers with regard to reducing PFAS concentrations below detection limits, the majority had 2 years or less of monitoring data, and the long-term impact of co-occurring compounds on barrier longevity is not yet known. Benzene and DRO have been shown to compete with PFAS for sorption sites and decrease equilibrium adsorption,^10,23^ but the ability of these hydrophobic compounds to displace previously adsorbed PFAS has not been assessed. Hydrophobic compounds associated with DRO are expected to impact PFAS sorption to CAC more than benzene, but the potential for DRO to cause PFAS desorption has not been evaluated. PFAS displacement and desorption by hydrophobic compounds must be quantified to properly manage in situ CAC barriers long-term and understand the potential for future PFAS release.

The impact of groundwater components on adsorption has been evaluated, but equilibrium adsorption measurements may not predict long-term performance when mass transfer rate-limited desorption exists in a system.^24^ Thus, it remains unclear how changing geochemical conditions may lead to previously sequestered PFAS being released from CAC. Studies evaluating PFAS release from CAC^25,26^ and anion exchange resin (AER)^25^ using artificial groundwater indicated that short-chain PFAS generally have lower retention on adsorbents compared to long-chain, which is consistent with their hydrophobic interaction capacities. However, these studies did not include hydrophobic components like DOM and DRO that can potentially displace previously adsorbed PFAS and lower the ability of the barrier to mitigate risk downgradient. The impact of these AFFF site-specific hydrophobic compounds on the performance of CAC barriers must be understood for proper remedy design and management.

The impact of PFAS headgroup and charge on their ability to be displaced from CAC by changing groundwater chemistry has not been investigated, but studies on PFAS desorption from soil provide some insights. Maizel et al.^27^ quantified the release of PFAS from an AFFF-impacted soil using artificial groundwater and found that neutral (based on predicted pK_a_) and zwitterionic PFAS (i.e., sulfonamides) were released more slowly than anionic PFAA. This behavior was attributed to more favorable interactions of neutral (more hydrophobic) and zwitterionic PFAS with negatively charged soil organic matter compared to those of anionic PFAA. Nickerson et al.^28^ examined the impact of pH and salt concentrations on PFAS desorption behavior from the AFFF-impacted soil. They observed that elevated pH (pH = 10) and salt concentrations (NaCl, I = 44.2 mM) increased the rate and extent of desorption for both anionic and zwitterionic PFAS from soil. They attributed this to soil organic matter having a higher density of negative charge at pH 10, but the effects of pH and salts could not be decoupled. These results for soil systems show that PFAS headgroup chemistry and IS both impact electrostatic surface interactions, but these desorption mechanisms have not yet been explored for CAC.

The present study aims to address uncertainty surrounding the long-term performance of in situ CAC by quantifying groundwater solute-induced desorption of PFAS from a commercial CAC (Intraplex, Germany) and providing insights into potential desorption mechanisms. Specific study objectives are to (1) quantify the extent and timing of PFAS release from CAC due to variations in influent water chemistry relevant to AFFF-impacted sites, particularly DRO and DOM, and (2) improve understanding of the mechanisms of PFAS displacement by relating PFAS structural characteristics to desorption behavior under varying influent compositions. The release of four PFAS from CAC for seven influent chemistries is quantified: control (1 mM NaHCO_3_, pH = 7.5), high and low IS, high- and low-MW DOM, aqueous-phase DRO, and DRO combined with high IS. We selected four representative PFAS that included both long- and short-chain perfluorocarboxylic acids, one short-chain perfluorosulfonic acid (PFSA), and one perfluoroalkane sulfonamide (FASA). FASA are a unique PFAS class that includes both neutral (protonated) and anionic (deprotonated) forms at near-neutral pH. To our knowledge, this is the first study to use flow-through columns to evaluate PFAS displacement from CAC for a range of influent chemistries and PFAS classes that are representative of AFFF-impacted sites. This work is particularly significant for coastal regions where multiple CAC barriers have been installed, and tidal pumping and seawater intrusion may lead to enhanced release of PFAS from barriers due to rapid changes in groundwater chemistry.^5^ Data presented informs future longevity assessments by providing insight into the potential for PFAS release and supports practitioners in long-term remedy management strategies.

## 2 Materials and Methods

### 2.1 Materials

Perfluorooctanoic acid (PFOA), perfluoropentanoic acid (PFPeA), and perfluorobutanesulfonic acid (PFBS) were purchased from TCI America (Portland, OR), and perfluorobutane sulfonamide (FBSA) was purchased from Cayman Chemical (Ann Arbor, MI). Stock solutions were prepared in Milli-Q water for use in adsorption experiments and in basic methanol (MeOH) for calibration standards. All stock solutions were stored in polypropylene (PP) vessels at ∼4 °C. Mass-labeled PFAS standards (13C8-PFOS, 13C8-PFOA, and 13C5-PFHxA) were purchased from Wellington Laboratories (Guelph, Canada). LC-MS/MS grade MeOH and ammonium acetate were purchased from Fisher Scientific. Organic matter was purchased from the International Humic Substances Society (IHSS); Suwanee River natural organic matter (SRNOM, IHSS reference 2R101N and 1R101N) and Elliott Soil fulvic acid (ESFA). SRNOM represents smaller-MW DOM (weight-average MW = 0.63–23 kg/mol), and ESFA represents larger-MW DOM (weight-average MW = 85 kg/mol).^29–31^ Detailed DOM MW distributions are listed in Table S1. DOM stock solutions were prepared by dissolving dry powder at 2 g/L in DI water overnight prior to filtration through a 0.45 μm nylon filter following previously described protocols.^29,30^ Though not directly measured here, Louie et al. determined the mass recovery of DOM in the filtrate of a 0.22 μm syringe filter. They found that the mass recovery was 94–96% for the same DOM types evaluated here, which indicates concentrations by weight (i.e., mg/L) should be similar for each stock solution used here.^30^ Automotive diesel was purchased from a commercial gas station in Pittsburgh, PA. Dry CAC (Intraplex, Germany) was provided by Intrapore and has been previously characterized by our group.^10,11^ Quartz sand was purchased from Thermo Fisher Scientific (Waltham, MA) and cleaned following previously described methods.^32^

### 2.2 Preparation of Diesel Water-Soluble Fraction (WSF)

A 2 L glass bottle was filled with 1600 mL of 1 mM NaHCO_3_ adjusted to pH 7.5 and a magnetic stir bar. 400 mL of automotive diesel was slowly added to avoid mixing. The water was stirred at 120 rpm at 25 °C for 2 weeks to achieve equilibrium based on previously described protocols.^33,34^ The slow-stirring method is a technique used to maintain the diesel-water interface and avoid formation of emulsions, which would supersaturate the aqueous phase.^33,35^ After mixing, the diesel phase was removed, and the aqueous phase was analyzed for DRO (C_10_–C_28_) by an environmental testing laboratory (Eurofins Environmental Testing, Lancaster, PA) using method 8015D (SW-846).^36^ Additional analytical details are provided in the SI.

### 2.3 CAC Preparation

PFPeA, PFBS, FBSA, and PFOA were all preadsorbed to CAC in a batch system based on previously described protocols.^37^ Briefly, 50 mL of 60 μM of each PFAS was prepared in 1 mM NaHCO_3_, pH = 7.5, and stirred for 5 min. 100 mg of CAC was added to the vessel, and the solution was mixed for 3 days on a rotary shaker, which had previously been shown as an adequate time to achieve adsorption equilibrium.^10,11^ After mixing, the solution was transferred to a beaker and dried at 80 °C until all water had evaporated, and CAC mass recovery was recorded. To confirm the mass of PFAS adsorbed, aqueous samples were collected before adding CAC and after 3 days of mixing and analyzed via isotope dilution. PFAS mass loadings were selected to be below the loading capacity of the CAC based on our previously published work with the same material. Loadings were selected to minimize PFAS–PFAS interactions relative to PFAS-CAC interactions and to avoid drying artifacts. In single-solute systems, maximum PFAS loadings based on the Langmuir isotherm model fits were between 170–290 μmol PFAS/g CAC. Here, individual PFAS loadings were between 25 and 32 μmol PFAS/g CAC.^11^ Dried CAC was combined with quartz sand at a 1% weight ratio, which is similar to field applications of CAC.^6^ Further details on CAC preparation can be found in Table S2.

### 2.4 Column Experiments

Glass columns (0.5 cm × 6.5 cm, Ace Glass, Vineland, NJ) were used along with coarse Unimin quartz sand at the inlet and outlet as support. Glass wool was also added to the column outlet to prevent the CAC displacement from the column. Preliminary tests confirmed that there was no PFAS adsorption to clean quartz sand, Unimin sand, and glass wool. The diameter of the quartz sand particles was less than 0.6 mm (30 mesh), which provided the minimum diameter of the column to particle to avoid serious wall effects and preferential flow paths.^38^ Sand porosity was determined by weighing dry-packed quartz, saturating with water, and weighing the quartz again.^39^ Column pore volume was based on the weight of sand added and calculated sand porosity.

Columns were prepared by semi-wet packing quartz sand, and the CAC-sand mixture generally followed guidelines of Lewis and Sjöstrom;^40^ thin layers of quartz sand (∼1.0 g total) and 1% CAC/sand (∼0.8 g total) were added to the column and were slowly water saturated from the bottom while gently tapping the column to remove air pockets.^40^ Columns could not be vibrated or vigorously agitated to avoid a large displacement of CAC from the sand. Table S3 includes detailed information on the packing of each column, and Figure S1 shows an example of a packed column. A piston pump (Fluid Metering, Inc. Lab Pump, Model QG 6) was used to pump influent into each column at a flow rate of 0.1 mL/min (288 pore volumes/day), resulting in a cross-sectional flow velocity of 7.3 m/d, resulting in a residence time of 5 min. This flow rate is higher than a normal pore water velocity but was selected to facilitate experimental timeframes and would represent a worst-case scenario for desorption, e.g., during tidal pumping events.^5,15^ Pumps were calibrated prior to each experiment.

Seven influent chemical conditions were evaluated with a shared background condition of 1 mM NaHCO_3_, pH = 7.5; control, high IS (100 mM, NaCl), low IS (10 mM, NaCl), SRNOM (10 mg/L, low MW), ESFA (10 mg/L, high MW), DRO (1400 μg/L), and DRO with high IS (DRO = 1400 μg/L, IS = 100 mM, NaCl). A negative control was also run following identical procedures with no PFAS preadsorbed onto CAC to ensure there were no detectable background levels of PFAS in the pumps or column system (data not shown). With the exception of the SRNOM (low-MW DOM) column, all column effluents were clear, and there was no notable remobilization of CAC. When influent containing SRNOM was used, early samples were light gray in color. To confirm there was no significant mass loss, an additional experiment was completed with SRNOM, where the mass of CAC in effluent was quantified and found to be <1% of the total CAC loaded into the column (data not shown). All columns were run in duplicate, except for ESFA and DRO, with high IS due to sample limitations.

### 2.5 Sample Preparation and PFAS Analysis

Composite effluent samples were collected for 5 h at regular time intervals and then once every 24 h for 5–10 days, depending on the experiment (Table S4). At each time interval, ∼1 mL of the composite effluent sample was immediately filtered through a 0.22 μm cellulose acetate syringe filter, which showed no significant adsorption of target PFAS in preliminary tests (data not shown). Filtered samples were stored in the dark at 4 °C until diluted and analyzed for PFAS as described below.

Analysis of all PFAS was performed using direct injection liquid chromatography tandem mass spectrometry (LC-MS/MS) using an Agilent 1100/6430 HPLC-QqQ with electrospray ionization (Agilent Technologies, US). PFAS were separated using gradient elution, detected with both quantitative and qualitative ion transitions, and quantified via external calibration (Table S5). Calibration curves (0.001–1.0 μM) were weighted (1/x), and correlation coefficients (*R* ^2^) were all >0.99 below 1 μM. Due to sample volume limitations (i.e., Samples 1–6 were <0.5 mL, Table S4), internal standards were only added to select duplicate samples from each column for quality control and to evaluate for matrix effects. Duplicate samples were analyzed via isotope dilution by spiking 30 μL of isotopically labeled PFHxA and PFOA into 970 μL of the composite effluent sample to achieve a final concentration of 18 μg/L (concentrations in μM are provided in Table S5). All duplicate samples analyzed via isotope dilution were within 20% of the same sample analyzed via external calibration. Additional analytical method details along with quality assurance and quality control protocols are described in SI. PFAS mass in each effluent sample was quantified as the product of measured sample volume and measured concentration. The fraction of mass released was then calculated based on the total amount of 1% CAC/sand added (Table S3) and the known amount of PFAS adsorbed to CAC (Table S2).

## 3 Results and Discussion

### 3.1 Effect of PFAS Structure on Release from CAC

PFAS structural characteristics were related to the total mass released in the control system (1 mM NaHCO3, pH = 7.5). Table 1 lists selected PFAS characteristics and the mass fraction released in the control column effluent based on the initial amount of PFAS preadsorbed to CAC (Tables S2 and S3). PFPeA had the largest fraction of mass released (83 ± 2%) after 1600 pore volumes, followed by PFBS (46 ± 4%), FBSA (30 ± 4%), and PFOA (1.9 ± 0.7%). Umeh et al. also observed very little release of PFOA and PFOS (<8%) during batch adsorption and desorption experiments in both Milli-Q water and artificial groundwater matrices.^41^ Minimal PFOA desorption was anticipated based on its longer perfluorinated alkyl tail and higher hydrophobicity relative to the three short-chain PFAS.^41^ Previous work from our group showed PFOA had higher adsorption affinity to the same CAC compared to other short-chain PFAA.^10,11^

**Table 1:**
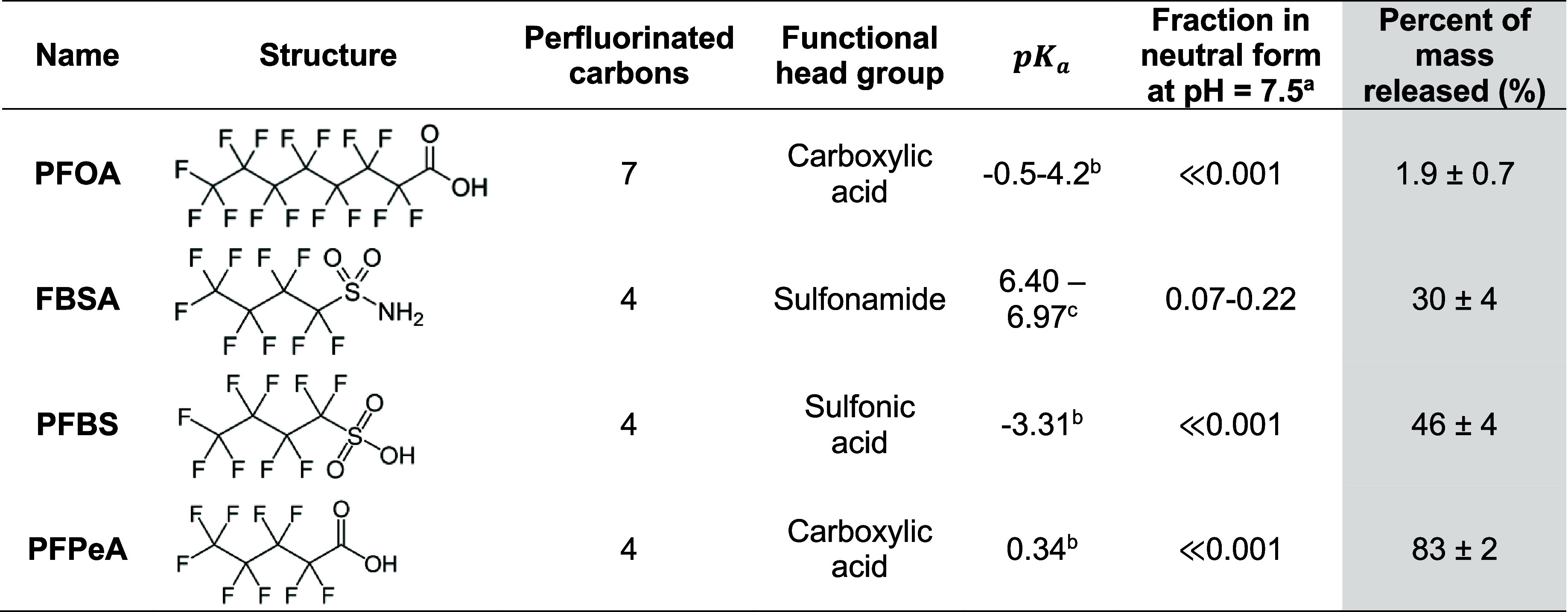
Selected Characteristics of PFAS Included in Column Studies and the Mass Fraction Released in Control Column Effluent after 1600 Pore Volumes Based on the Initial Amount of PFAS Preadsorbed to CAC

a Schwarzenbach et al.^42^

b Gagliano et al.^18^

c Murillo-Gelvez et al.^43^

Comparison of three PFAS with the same number of perfluorinated carbons (C4) but different functional headgroups revealed that headgroup chemistry impacts overall mass release, and weak acids have higher sorption stability (Table 1). Both PFPeA and PFBS are anions at environmentally relevant pH, whereas FBSA is a weak acid and will have a fraction present in the neutral form at pH = 7.5 (Table 1).^44,45^ A higher fraction of PFPeA was released in the control column effluent than PFBS, suggesting that PFCA are more susceptible to release from CAC than PFSA (Table 1). Niarchos et al. also observed faster breakthrough and desorption of PFCA compared to PFSA in dynamic column experiments with CAC.^26^ PFCA tend to have less favorable interactions with AC materials than PFSA with the same number of perfluorinated carbon atoms.^18,46^ FBSA had the lowest mass fraction released of the three short-chain PFAS, suggesting that the fraction of neutral species enhances sorption stability compared to anions (Table 1). pK_a_ values for FBSA have been estimated between 6.4 and 6.97, indicating that between 7 and 22% are present in the neutral form.^43^ Neutral species generally have lower water solubilities, which supports this observation.^42^

Interestingly, trends in the desorption behavior of FBSA and PFBS at pH 7.5 did not match trends in their adsorption behavior. Under the same control condition (1 mM NaHCO_3_, pH = 7.5), PFBS had higher adsorption affinity to CAC compared to FBSA (Table S6); however, less FBSA was released during column experiments compared to PFBS (Table 1). At pH = 7.5, the CAC used in this study has an acidic external surface (pH_IEP_ = 4.5) and an overall (internal + external) positive surface charge (pH_PZC_ of 9.5), and differences in each compound’s ability to undergo electrostatic and hydrophobic surface interactions may explain these observations.^11,47^ At pH = 7.5, anionic PFBS experiences both favorable electrostatic and hydrophobic surface interactions, whereas the fraction of neutral FBSA species (Table 1) can only undergo hydrophobic surface interactions with CAC. While the addition of favorable electrostatic interactions benefits adsorption for PFBS, hydrophobic surface interactions for FBSA appear to be more beneficial for sorption stability. Importantly, these results indicate that equilibrium adsorption parameters used for longevity modeling may not fully predict desorption potential in systems where mass transfer rate-limited desorption exists. To evaluate whether nonequilibrium conditions existed in the control system, additional analysis was completed (data shown in SI) where the aqueous effluent concentration was predicted using Freundlich isotherm parameters from previous work^11^ and compared to the measured effluent concentrations. The measured concentration of all PFAS was above what was predicted based on equilibrium adsorption at both early and later eluting pore volumes, confirming that mass transfer rate-limited conditions existed in the system.

### 3.2 Effect of DOM Molecular Weight on PFAS Release from CAC

Low-MW DOM (SRNOM1 and SRNOM2) in the column influent generally led to higher displacement of all PFAS compared to high-MW DOM (ESFA) and had a greater impact on the timing of mass release. Figure 1 shows effluent concentration profiles as a function of pore volume (time), where concentrations are normalized to the maximum observed effluent concentrations for the respective study to better illustrate trends between compounds and influent conditions. Therefore, the area under the curve is not representative of the total mass released. The percentage of the total mass released is shown in the upper right corner of each plot. Table S7 lists the percentage of total mass released for all column conditions. Full concentration profiles for both the normalized and raw effluent concentrations are presented in Figures S2–S5 and S6–S9, respectively. DOM MW distributions have previously been reported and are detailed in Table S1;^29,30^ SRNOM1 has a weight-averaged MW of 0.47 kg/mol, SRNOM2 has a weight-averaged MW of 23 kg/mol, and ESFA is 85 kg/mol, determined by size exclusion chromatography with multiangle light scattering (SRNOM1, ESFA)^29^ and vapor pressure osmometry (SRNOM2),^48^ respectively. Compared to the control, low-MW DOM increased the percent of PFOA mass released by 21%, FBSA by 11%, PFBS by 23%, and PFPeA by 6%; high-MW DOM increased the percent of PFOA mass released by ∼0%, FBSA by 3%, PFBS by 26%, and PFPeA by 3% (Table S7). Low-MW DOM also had a greater impact on equilibrium adsorption of PFAA to the same CAC, likely due to increased pore blockage and surface coverage.^11^ Previous work has shown that DOM can displace perfluorobutanoic acid (PFBA), another short-chain PFCA, from granular activated carbon (GAC) for drinking water applications.^19,46^ Here, the fraction of PFPeA mass released was not enhanced compared to the control, while PFBS and FBSA mass release was enhanced by DOM. One possible explanation is that 83% of PFPeA was released in the absence of DOM (Table 1) and the remaining ∼17% is likely adsorbed to CAC micropores, where either DOM displacement or PFPeA desorption was limited by time scales of diffusion.

**Figure 1:**
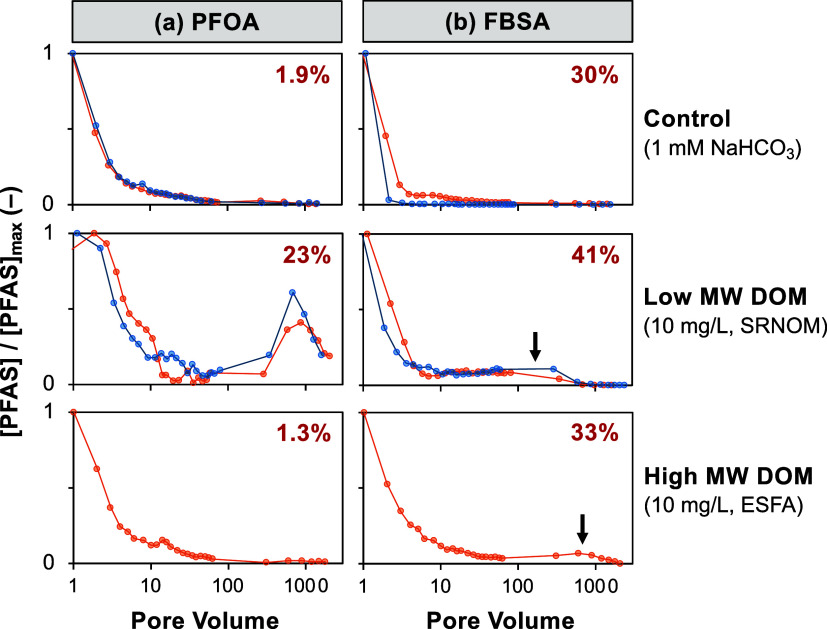
Maximum value normalized effluent concentrations versus pore volumes for (a) PFOA and (b) FBSA due to influent dissolved organic matter (DOM). Influent conditions are noted on the right, and the percent of total mass released for each condition is in the upper right corner of each plot. Black arrows in panel (b) highlight the changing impact of the DOM with different molecular weights. Blue and orange data points in each panel represent replicate experiments. Note for low-MW DOM, orange data points represent SRNOM1 and blue data points represent SRNOM2. Other short-chain PFAS behaved similarly to FBSA and are shown in SI (Figures S2–S3).

DOM also caused a secondary release of each PFAS at later pore volumes (time), and differences in DOM MW led to differences in the timing of mass release (Figure 1). Low-MW DOM (SRNOM) caused more PFAS mass release at earlier pore volumes than high-MW DOM (ESFA). The most significant difference between DOM types was observed for PFOA; low-MW DOM readily displaced PFOA, leading to a significant spike in concentration (50–60% of the maximum) around 700 pore volumes, while high-MW DOM did not (Figure 1a). Low-MW DOM has been shown to have higher rates of diffusion through water than high-MW DOM, which could explain these observations.^49^ The effluent profile of FBSA and other short-chain PFAS (Figures S2–S3) visually represents this effect (arrows in Figure 1b); low-MW DOM caused effluent concentrations to increase from 100 to 400 pore volumes, while high-MW DOM led to a diffuse increase in effluent concentrations between 350 and 1000 pore volumes. This observation is also illustrated in Figure S10, which shows the fraction of total PFAS mass released during distinct pore volumes. Notably, for the three short-chain PFAS, the fraction of mass released increases at earlier pore volumes when low-MW DOM is present in column influent compared to high-MW DOM (Figure S10). There could also be subtle differences in the molar concentrations of each DOM stock solution. Solutions were prepared by weight of dry DOM, and MW differences will affect the number of DOM “molecules” present in solution; low-MW DOM will have more molecules in a 10 mg/L solution than high-MW DOM at 10 mg/L, which may enhance observed effects. Additional mechanistic discussion is included in Section 3.5.

### 3.3 Effect of Ionic Strength on PFAS Release from CAC

Elevated IS impacted both the extent and rate of short-chain mass release more significantly than long-chain PFOA (Figure 2 and Table S7). Influent IS was modified using NaCl to achieve a low (10 mM) and high (100 mM) condition, which would be representative of a noncoastal and coastal site, respectively.^5^ PFOA release decreased from 1.9% in the control to 0.9% and 0.7% for high and low IS, respectively, but neither were statistically different from the control (*p* < 0.05, Figure 2a, and Table S7). In equilibrium batch tests, PFOA adsorption was minimally enhanced in the presence of high IS (100 mM, NaCl), likely due to a combination of salting-out effects and electric double layer compression, which both increase hydrophobic interaction capacity.^11^ Umeh et al. also found minimal desorption of PFOA from powdered activated carbon (PAC) in the presence of inorganic salts.^41^ Lower release of PFOA in effluent at nonequilibrium conditions in column studies here suggests that stronger hydrophobic interactions indeed enhance the sorption stability of long-chain PFAS even in the presence of salts.

**Figure 2:**
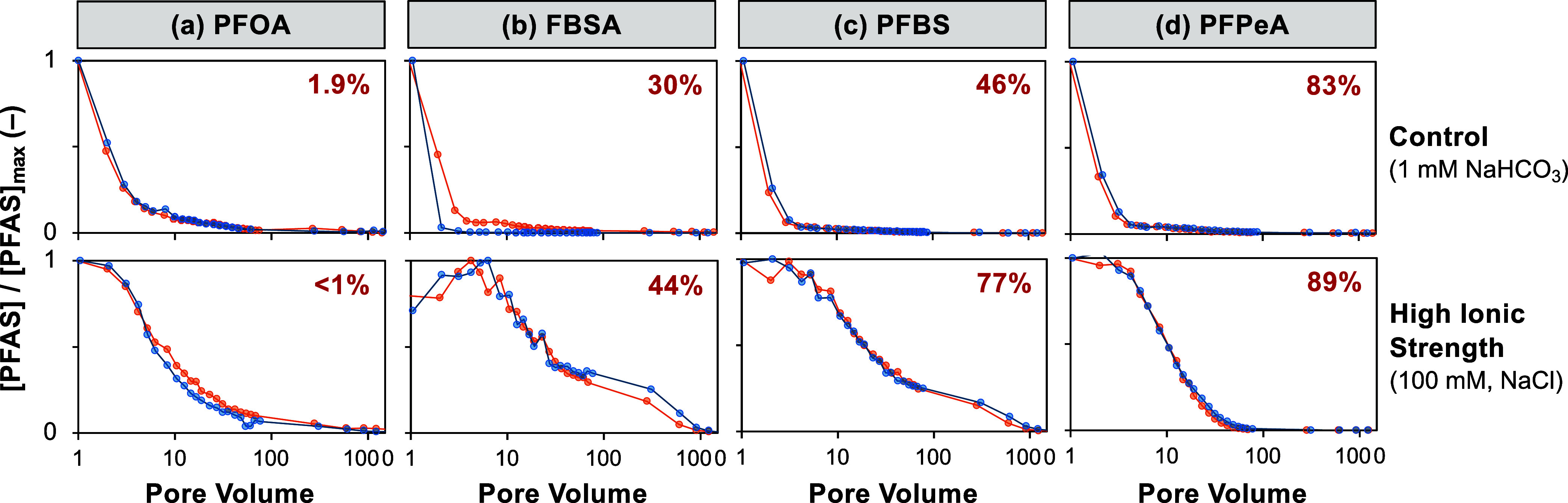
Maximum value normalized effluent concentrations versus pore volumes for (a) PFOA, (b) FBSA, (c) PFBS, and (d) PFPeA due to increased ionic strength. Influent conditions are indicated on the right, and the percent of total mass released for each condition is in the upper right corner of each plot. Blue and orange data points in each panel represent replicate experiments. Effluent profiles for low IS are shown in Figures S2–S5.

Both high (100 mM) and low (10 mM) IS increased the mass release of all three short-chain PFAS compared to the control, with the magnitude of effect greater for high IS and dependent on functional headgroup (Figure 2 and Table S7). Compared to the control, high IS increased the displacement of FBSA by 14% (Figure 2b), PFBS by 32% (Figure 2c), and PFPeA by 6% (Figure 2d). Inorganic ions disrupt the electrostatic surface interactions between anionic PFAS and the positively charged CAC surface (pH_PZC_ = 9.3), and specific headgroup chemistry will impact this interaction. We expect inorganic ions to impact the release of PFPeA and PFBS more than FBSA because of their negatively charged headgroups; at pH = 7.5, both PFBS and PFPeA are completely ionized, whereas a fraction of FBSA is present in neutral form (Table 1). Previous batch isotherm tests showed that when IS was increased to 100 mM compared to the control of 1 mM NaHCO_3_, values of *K*_d_ decreased by 1 order of magnitude for PFPeA and by a factor of 3 for PFBS.^11^ High IS caused the fraction of PFBS mass released to increase more than FBSA, which is consistent with a fraction of the sorbed neutral FBSA being less affected by inorganic anions; however, PFPeA mass release was not as enhanced in contrast to results observed during equilibrium adsorption studies.^11^ This is likely due to PFPeA having low mass retention on CAC even in the control system (∼17% retained), but highlights another scenario where equilibrium adsorption did not predict desorption potential in a mass transfer rate-controlled system.

Elevated IS also decreased the rate of PFAS mass release, particularly for the three short-chain PFAS (Figure 2). Mass release of the three short-chain PFAS was extended up to 1000 pore volumes. While the total mass of PFOA released was less affected than the short-chain PFAS, the rate of release was also decreased (Figure 2). High IS caused a greater effect than low IS, indicating that inorganic ions can influence PFAS release profiles in a concentration-dependent manner (Figures S2–S4). Specific mechanisms causing this observed effect are discussed in Section 3.5.

### 3.4 Effect of DRO on PFAS Release from CAC

DRO caused the highest displacement of PFOA but had less overall impact on the release of short-chain PFAS (Figure 3 and Table S7). DRO in the column influent increased the fraction of PFOA mass released from less than 1% in the control to 67% (Figure 3a and Table S7). DRO compounds are hydrophobic and can displace PFOA from the CAC surface, which largely adsorbs through hydrophobic surface interactions. DRO is also more hydrophobic than DOM, which is likely why enhanced PFOA release was observed for DRO compared to low-MW DOM. DRO caused the release of the three short-chain PFAS to increase, but none were statistically greater than the control (*p* < 0.05), and the magnitude of change was less than for PFOA. Compared to the control, PFPeA mass release was unaffected, PFBS displacement increased by 26%, and FBSA displacement increased by 6% when DRO was in the influent (Table S7). When both DRO and high IS were combined in influent, the fraction of mass released was nearly identical to DRO alone for each PFAS, indicating that hydrophobic displacement by DRO is the predominant desorption mechanism (Table S7 and Figures S2–S5).

**Figure 3:**
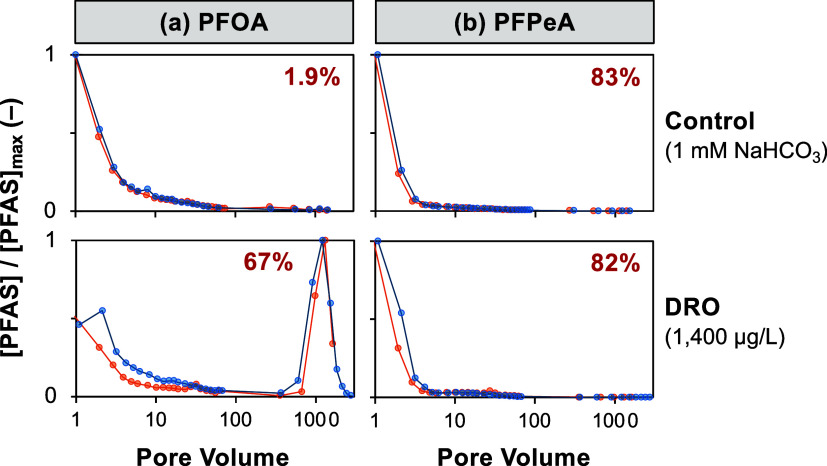
Maximum value normalized effluent concentrations versus pore volumes for (a) PFOA and (b) PFPeA due to influent diesel range organics (DRO). Influent conditions are indicated on the right, and the percent of total mass released for each condition is in the upper right corner of each plot. Blue and orange data points in each panel represent replicate experiments. Other short-chain PFAS behaved similarly to PFPeA and are shown in SI (Figures S2 and S4).

DRO had the most significant impact on the temporal change of PFOA mass release, while the effluent concentration profiles of the three short-chain PFAS were less affected (Figures 3 and S2–S5). DRO caused a significant increase in PFOA effluent concentrations from 600 to 2500 pore volumes, with a maximum concentration nearly double that of early pore volumes. This was also the only experimental condition where the maximum concentration of PFOA did not occur in the first several pore volumes, and nearly half of the PFOA mass is released after 1000 pore volumes (Figure 3a). The desorption profile was also similar to that of low-MW DOM (Figure 1a), suggesting similar mechanisms of displacement. The mass fraction of short-chain PFAS released, being less impacted by DRO, was a somewhat unexpected result and emphasizes the relative importance of surface-interaction mechanisms. DRO could be viewed as a probe to assess the importance of hydrophobic surface interactions, and its relative impact on the overall release depends on the hydrophobicity of the individual PFAS. Short-chain PFAS such as PFBS and PFPeA are less affected by DRO than PFOA because they are less hydrophobic. Mechanisms of PFAS displacement by DRO are discussed in Section 3.5.

### 3.5 Proposed Mechanisms of Desorption and Conceptual Model

On the basis of the results discussed above, we propose a conceptual model that is consistent with the potential mechanisms by which IS, DOM, and DRO impact the extent and temporal change of PFAS mass release from CAC (Figure 4). Previous characterization by our group revealed that Intraplex CAC has a heterogeneous distribution of surface charge, where positive charges are located on internal surfaces (i.e., micropores) and the external CAC surface is negatively charged.^10,11^ Therefore, we believe short-chain PFAS adsorb primarily to internal CAC micropores due to favorable electrostatic interactions, whereas long-chain PFAS will be enriched at CAC outer surfaces through hydrophobic surface interactions.^11^ Short-chain PFAS also have higher molecular diffusivities than long-chain PFAS and can more readily diffuse into CAC micropores.^46^ Differences in where PFAS adsorb to the CAC surface have implications for desorption mechanisms and are consistent with the results reported here.

**Figure 4:**
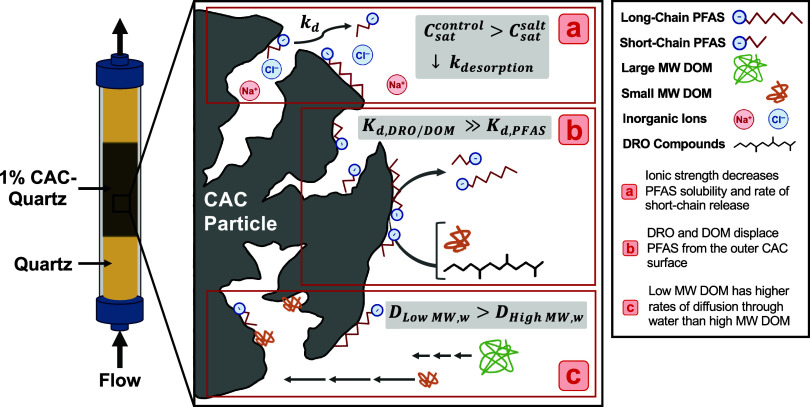
Conceptual model illustrating potential mechanisms of PFAS interaction with the CAC surface and impact of influent variables. Mechanisms represented are (a) decreased rate of desorption due to elevated ionic strength, (b) hydrophobic displacement by DRO and DOM, and (c) differences in rates of DOM molecular diffusion due to different molecular weights.

The electrostatic interactions between PFAS and CAC will be affected by elevated IS with the magnitude of effect dependent on PFAS hydrophobicity. High IS decreased the total mass of PFOA released, increased the total mass of PFBS released, and only slightly increased the total mass of FBSA and PFPeA released. The most notable impact IS had on PFAS release was decreasing the rate of desorption of short-chain PFAS (Figure 2). One potential explanation is a decrease in the rate of mass transfer due to lower aqueous solubility (i.e., *C*_sat_) in the presence of salts, i.e., the salting-out effect (Figure 4a), which would slow mass transfer from CAC to water.^42,50^ Additionally, inorganic anions may be displacing PFAS from CAC micropores, which would require adequate time for diffusion out of the pore. The flow rate used in this study is such that residence times are well below the time required to reach CAC-water equilibrium; in our previous work, equilibrium is achieved between 24 and 72 h^11^ whereas column residence time is around 5 min. The elevated flow rates used in this study create the greatest concentration gradient to promote mass transfer from CAC to the water phase. However, this rate-controlled process is slowed if PFAS solubility in water is decreased. Multiple studies have reported higher adsorption of long-chain PFAS to various adsorbents in the presence of salt in batch systems, hypothesizing that salt decreases PFAS solubility in water, thus promoting sorption to solids.^13,18,50,51^ These effects also slow the desorption rate of short-chain PFAS from CAC. The impacts of this mass transfer rate-limited process are diminished for barriers with longer groundwater residence times, where equilibrium sorption/desorption may be achieved. The importance of these effects will depend on the hydraulic conditions at the site.

A competitive displacement mechanism by hydrophobic influent constituents is proposed based on the delayed release of PFAS by influent containing DOM and DRO (Figure 4b). Initial PFAS loadings were selected to be below the loading capacity of the CAC based on our previous work with the same material.^10,11^ During early eluting pore volumes, influent DOM or DRO is likely adsorbing to available sorption sites, and once excess sites are occupied, DRO or DOM will displace adsorbed PFAS because of its higher affinity for the carbon surface (i.e., *K*_d,DRO/DOM_ ≫ *K*_d,PFAS_). Previous studies with the same material indicate that the CAC has a maximum capacity between 170 and 290 μmol/g CAC in single-solute systems (PFOA-PFBS, average of 188 μmol/g).11 Here, mass loading on CAC was around 30 μmol/g for each PFAS (113 μmol total PFAS/g CAC). Therefore, it is estimated that around 30–60% of the CAC’s PFAS sorption capacity is still available after initial PFAS loading.^11^ The significantly enhanced displacement of PFOA in the presence of DRO and low-MW DOM was a unique outcome that was not observed for the three short-chain PFAS. The location of PFAS sorption to the CAC surface is one potential explanation as to why DRO or DOM did not enhance short-chain displacement as drastically as PFOA.

We propose a conceptual model where PFOA and DRO are competing for the same sorption sites, whereas short-chain PFAS sorbed in CAC micropores are less affected by competing hydrophobic compounds (Figure 4b). Previous CAC characterization and work by our group suggest that short-chain PFAS are enriched on inner pores due to electrostatic affinity for positively charged surfaces located in micropores, as well as their higher diffusion rates through water.^11,46^ Conversely, PFOA and other hydrophobic compounds like DRO and DOM adsorb on CAC outer surfaces because of their increased hydrophobicity. Therefore, hydrophobic compounds such as DOM and DRO are not competing for the same sorption sites as short-chain PFAS. Because of sample volume limitations, a drawback of the present study is a lack of DRO and DOM measurements in column effluent samples to confirm this likely mechanism of displacement. Nevertheless, results show that co-occurring hydrophobic compounds such as DRO and DOM, which are often found at many AFFF-impacted sites, can displace previously adsorbed PFAS from CAC, including longer-chain PFAS like PFOA.

Low-MW DOM caused increased mass release of all PFAS compared to high-MW DOM and at earlier pore volumes, which may be a result of higher rates of diffusion to the CAC surface and increased concentration of DOM molecules (Figure 4c). Displacement of adsorbed PFAS by low-MW DOM was observed earlier than by high-MW DOM because low-MW DOM has faster mass transfer kinetics.^49^ Release of short-chain PFAS was also not as significantly enhanced by low-MW DOM as PFOA, which is in contrast to batch isotherm data, where DOM had a lower impact on PFOA sorption compared to short-chain PFAS.^11^ In that work, DOM was combined with CAC for 3 days prior to the addition of target PFAA, which allows DOM molecules to diffuse and adsorb to the CAC surface and thus inhibit PFAS adsorption to a higher extent. In the present study, slow DOM diffusion to the CAC surface and into pores reverses this result. The time scales for DOM diffusion to the CAC surface can be estimated using reported diffusion coefficients in water and an assumed CAC boundary layer (eq 1)­

1 $$\mathrm{time}\;\mathrm{for}\;\mathrm{diffusion}\;\mathrm{to}\;\mathrm{the}\;\mathrm{CAC}\;\mathrm{surface}=\frac{{\left(L\right)}^{2}}{{2\times D}_{\mathrm{DOM},w}}$$

where *L* is the boundary layer thickness (cm) and *D*_DOM,w_ (cm^2^/s) is the diffusion coefficient of DOM in water. Balch and Guéguen^49^ reported the diffusion coefficients for several DOM types based on molecular weight were on the scale of 10^–7^–10^–8^ cm^2^/s, and a typical boundary layer for particles in water is around 200 μm.^42^ Therefore, time scales for diffusion to the CAC surface are estimated to be on the scale of 30–300 min. The column retention time is in this study is 5 min, and the diffusion of DOM into CAC micropores is a mass transfer rate-limited process. Based on both equilibrium studies and flow-through columns, overall mass release into column effluent due to DOM may increase with longer contact times, allowing adequate time for diffusion.

## 4 Implications for CAC Barrier Longevity

As the use of in situ CAC adsorptive barriers continues to grow, particularly in coastal regions where groundwater velocities are higher, and the water chemistry can rapidly change due to tidal pumping, understanding whether and when previously sequestered PFAS will be released is vital for technology implementation and long-term monitoring plans. To our knowledge, this study is the first flow-through column assessment of PFAS desorption from CAC under various influent conditions. Comparison of four representative PFAS showed that long-chain PFAS are less susceptible to desorption than short-chain PFAS. Results from the three short-chain PFAS also reveal that headgroup chemistry will dictate desorption behavior and that PFCA is most susceptible, followed by PFSA and FASA. FASA are unique PFAS due to their sulfonamide functional group and high pK_a_ values, which cause a small fraction to be present in the neutral form at environmentally relevant pH.

Elevated IS only slightly enhanced the total mass release of short-chain PFASs but did impact the rate of desorption. Specifically, high IS (100 mM) caused all three short-chain PFAS to be released more slowly from CAC, which may become significant at coastal groundwater sites where CAC barriers will experience saltwater intrusion.^5^ PFAS displacement by influent DRO and low-MW DOM indicates that hydrophobic organic compounds have the potential to cause secondary releases of previously adsorbed PFAS. Notably, DRO caused a significant mass release of PFOA, indicating that more hydrophobic solutes may induce the desorption of PFOA and shorter-chain PFAS. This effect has been observed in flow-through columns with GAC, where short-chain PFAS, such as PFBA, were displaced by longer-chain PFAS.^46^ These results suggest the potential for longer-chain and more hydrophobic PFAS, such as PFOS, to displace PFOA. This should be explored further, as this effect is highly relevant to AFFF-impacted sites where PFOS typically dominates the PFAS profile due to historical formulations of AFFF.^52^ Barrier design is often driven by the behavior of PFOA due to its extremely low maximum contaminant levels (MCL) set by the US EPA in April 2023 (4.0 ng/L), and any displacement of PFOA caused by DRO, DOM, or more hydrophobic PFAS could lead to regulatory exceedances.^2^ Future work should quantify the adsorption of DRO and DOM to CAC both in batch (equilibrium) and dynamic (mass transfer rate-limited) studies to better understand the mechanisms of PFAS displacement.

To date, CAC barrier longevity assessments have been based on equilibrium adsorption parameters,^5–7,53^ but the present study demonstrates that equilibrium partitioning may not capture PFAS desorption and displacement by coexisting groundwater solutes when mass transfer rate limitations exist. The relatively high pore water velocity used in this study (7.3 m/d) results in a residence time of 5 min, in contrast to the 24–72 h required for equilibrium sorption based on previous batch tests with the same material.^11^ This short residence time amplifies mass transfer rate-limited desorption effects relative to those occurring in slower-flowing groundwater systems. At sites with slow-moving groundwater, residence times in a CAC barrier may represent equilibrium, and the effects observed here at high flow rates may be moderated. A database was recently published detailing the CAC barrier dimensions and groundwater flow conditions at 17 sites where CAC barriers have been installed. Median barrier width was 3.96 m, and the median groundwater flow velocity was 0.035 m/d, resulting in a median residence time of 113 days.^14^ Another a recent evaluation of CAC barrier performance in coastal regions showed that near-shore groundwater velocity can fluctuate between 0.1 and 0.36 m/d due to tidal pumping.^5^

While mass transfer rate limitations may not always be expected at field sites with slow-moving groundwater, they are present in many water-treatment systems and potentially present in some groundwater systems. Thus, it remains important to understand the properties of influent groundwater that may induce the displacement or desorption of previously sequestered PFAS. Our results also suggest that accurate modeling of CAC performance may need to consider mass transfer rate-limited sorption/desorption when groundwater retention times are lower than 24 to 72 h. The work presented here provides both fundamental and practical insights into PFAS behavior in CAC barriers; results also enhance understanding of PFAS displacement mechanisms from CAC that can be used to inform future groundwater modeling, barrier design, and long-term site management strategies.

## Supplementary Material



## Notes

Analytical method details; quality assurance and quality control details; DOM molecular weight distributions; details of CAC-sand preparation; column packing details; column effluent sample collection details; LC-MS (QqQ) operating details and method detection limits; relevant Freundlich isotherm parameters; mass fraction of PFAS released for each column condition; example of fully packed saturated column; and all effluent concentration profiles (PDF)
