# Supplementary material for: Groundwater Solute-Induced Desorption of Perfluoroalkyl Substances (PFAS) from Colloidal Activated Carbon (CAC)
Source: Environ Sci Technol. 2026 Jun 1;60(23):16866–76. doi: 10.1021/acs.est.6c00984 (PMC13276901; doi:10.1021/acs.est.6c00984)
Supplement: Supplementary file 1 [file es6c00984_si_001.pdf]

Supporting Information for

Groundwater solute-induced desorption of perfluoroalkyl substances (PFAS) from colloidal activated carbon (CAC)

Rachel A. Molé, [a] Adriana C. Velosa, [a] Xitong Liu,[b] Guangbin Li,[c] Dimin Fan,[d] Anthony Danko,[e] and Gregory V. Lowry [a] *

[a] Department of Civil and Environmental Engineering, Carnegie Mellon University, Pittsburgh, PA, 15213, USA

[b] Department of Civil and Environmental Engineering, The George Washington University, Washington, DC, 20052, USA

[c] Department of Civil and Environmental Engineering, University of Maryland, College Park, MD, 20742, USA

[d] Geosyntec Consultants, Inc, 10211 Wincopin Cir Floor 4, Columbia, MD, 21044, USA

[e] Naval Facilities Engineering Systems Command, Engineering and Expeditionary Warfare Center, Port Hueneme, CA, 93043, USA

*Corresponding Author

E-mail address: glowry@cmu.edu (G.V. Lowry)

Tel: (412) 268-2948, Fax: (412) 268-7813

Carnegie Mellon University, Porter Hall 119, 4815 Frew St., Pittsburgh, PA, 15213-3890

The file includes:

<u>Number of pages:</u> 19

<u>Number of tables:</u> 7

<u>Number of figures:</u> 10

Table of Contents

PFAS Analytical Methods 3

PFAS Quality Assurance and Quality Control 3

Analysis of Diesel Water Soluble Fraction (WSF) 3

Analysis of Non-Equilibrium Conditions in the Control Column 4

Table S1. DOM molecular weight distributions. 5

Table S2. CAC-Sand preparation details. 5

Table S3. Column packing details. 6

Table S4. General timing of composite effluent sample collection. 6

Table S5. Optimized MS/MS transitions, QqQ operating parameters, method detection limits (MDL) and corresponding isotopically labeled standards (ILS) for each PFAA 7

Table S6. Freundlich adsorption isotherm parameters collected following protocols described in previous work 7

Table S7. Mass fraction of PFAS released in column effluents after 1,600 pore volumes for each influent condition based on the initial amount of PFAS pre-adsorbed to CAC. 8

Figure S1. Column packed with clean quartz sand and 1% CAC/sand mixture with coarse Unimin sand at the inlet and outlet and glass wool at the outlet. 9

Figure S2. PFPeA full maximum value normalized effluent concentration profiles. 10

Figure S3. PFBS full maximum value normalized effluent concentration profiles. 11

Figure S4. FBSA full maximum value normalized effluent concentration profiles. 12

Figure S5. PFOA full maximum value normalized effluent concentration profiles. 13

Figure S6. PFPeA full maximum value raw effluent concentration profiles. 14

Figure S7. PFBS full maximum value raw effluent concentration profiles. 15

Figure S8. FBSA full maximum value raw effluent concentration profiles. 16

Figure S9. PFOA full maximum value raw effluent concentration profiles. 17

Figure S10. Fraction of total PFAS mass recovered in distinct pore volume bins for each influent chemistry condition. 18

PFAS Analytical Methods

To separate background PFAS present in the HPLC pumps from sample peaks, a C_{18} delay column (ZORBAX RR Eclipse Plus, 4.6 x 50 mm, 95 Å, 3.5 µm, Agilent Technologies, US) was installed between the binary pumps and autosampler. A C_{18} analytical column (Poroshell 120 EC-C18, 3.0 x 50 mm, 2.7 µm, Agilent Technologies, US) was used for analyte separation with the column temperature maintained at 40 °C. Injection volumes were 20 µL. Mobile phases were A: 20 mM ammonium acetate in Milli-Q water and B: MeOH with a flow rate of 0.4 mL/min. Mixtures of PFAS were separated using gradient elution. PFAS were detected with multiple reaction monitoring using quantitative and qualitative ion transitions (Table S4) and quantified via both external calibration and isotope dilution.

PFAS Quality Assurance and Quality Control

Laboratory reagent blanks and controls were run along with continuing calibration verifications (CCVs) at least every 10 samples and were required to be within 20% of the expected concentration to ensure calibration validity throughout sample runs. For each column, blank influent samples were collected and analyzed. Due to the bench scale nature of the work and high PFAS concentrations, isotope dilution and internal standards were not utilized for every sample and only selected samples from each column were spiked with internal standards and analyzed via isotope dilution. Samples analyzed via isotope dilution were required to be within 20% of samples measured via external calibration. Matrix matched samples were also evaluated to ensure that elevated ionic strength, DOM, and DRO did not impact instrument sensitivity. Method detection limits (MDL) were determined using the EPA MDL Procedure, Revision 2. In brief, 10 spiked samples three times the estimated MDL were prepared along with 10 method blank samples. Method blanks returned no numerical results, so the MDL was calculated as the standard deviation of the 10 prepared samples multiplied by $t(9, 0.99) = 2.821$. MDLs are presented in Table S5.

Analysis of Diesel Water Soluble Fraction (WSF)

After the aqueous phase was separated from the diesel phase, it was diluted by half with laboratory Milli-Q waster and sent to Eurofins Lancaster Laboratories Environmental Testing for analysis. Diesel range organics (DRO) corresponds to the range of alkanes from C10 to C28 and covering a boiling point range of approximately 170ºC - 430ºC. DRO were quantified via EPA method 8015D (SW-846). The final DRO concentration was 3,500 µg/L (reporting limit = 110 µg/L). The stock DRO WSF (7,000 µg/L) was used to prepare the column influent conditions described in Section 2.4.

Analysis of Non-Equilibrium Conditions in the Control Column

Additional analysis was completed where the aqueous effluent concentration was predicted using Freundlich isotherm parameters and compared to the measured effluent concentration. This analysis was completed for both the first eluting pore volume and the ~50th eluting pore volume of the control system based on the visual observation that the initial mass release subsides after 50 pore volumes (Figures S2 – S9).

Below is a summary of the results from that analysis where Freundlich adsorption parameters are from our previous study with the same CAC material[1], C_{solid} was determined based on CAC preparation described in Table S2 and PFAS mass balance at the 10th pore volume, predicted $C_{effluent}$ was derived using Freundlich parameters, and measured $C_{effluent}$ was from the first measured pore volume and 50th measured pore volume.

	PFAS	K_F $[\mu mol^{1-n} L^n g^{-1}]$	n	C_{solid} $\left[\frac{\mu mol}{g\ CAC}\right]$	Predicted $C_{effluent}$ $[\mu M]$	Measured $C_{effluent}$ $[\mu M]$
First Pore Volume	PFOA	181.97	0.19	28.8	0.000061	0.27
	PFPeA	50.12	0.54	32.1	0.44	149.61
	PFBS	93.33	0.32	25.3	0.017	19.15
	FBSA	64.57	0.42	27.0	0.13	10.95
50th Pore Volume	PFOA	181.97	0.19	28.70	0.000060	0.017
	PFPeA	50.12	0.54	13.70	0.091	1.16
	PFBS	93.33	0.32	22.34	0.011	0.26
	FBSA	64.57	0.42	25.21	0.11	0.18

Table S1. DOM molecular weight distributions.

NOM Type	*Mass distribution of molecular weight* > 100 kg/mol (%)	50 – 100 kg/mol (%)	10 – 50 kg/mol (%)	< 10 kg/mol (%)	Weight-Average MW
**ESFA***	3.0	1.1	58	38	85 kg/mol
**SRNOM1***	1.3	1.1	37	61	23 kg/mol
**SRNOM2****	*Data not provided*				0.63 kg/mol

*Data reproduced from Louie et al. (2015)[2]
**Data reproduced from Pavlik and Perdue (2015)[3]

Table S2. CAC-Sand preparation details. Two batches of CAC were used throughout the study.

Batch #1: 107.3 mg of dried CAC was added to 10.73 g of quartz sand	$C_{aq}^{initial}[\mu M]$	$C_{aq}^{final}[\mu M]$	**% Adsorbed**	$\frac{\mu mol\ PFAS}{mg\ CAC}$
**PFOA**	62.44	0.12	0.99	0.032
**PFBS**	54.90	0.49	0.99	0.027
**FBSA**	58.55	0.44	0.99	0.025
**PFPeA**	69.69	3.60	0.95	0.029

Batch #2: 89.4 mg of dried CAC was added to 8.94 g of quartz sand	$C_{aq}^{initial}[\mu M]$	$C_{aq}^{final}[\mu M]$	**% Adsorbed**	$\frac{\mu mol\ PFAS}{mg\ CAC}$
**PFOA**	60.08	0.001	0.99	0.027
**PFBS**	52.73	0.018	0.99	0.024
**FBSA**	60.15	0.020	0.99	0.027
**PFPeA**	68.57	0.19	0.97	0.031

Table S3. Column packing details.

Experiment	Quartz sand (g)	1% (wt/wt) CAC/Quartz (g)	Pore Volume (mL)
Control_R1	1.1604	0.8042[1]	0.512
Control_R2	0.9977	0.8004[1]	0.468
Control_R3	1.1037	0.8002[1]	0.496
Low Ionic Strength_R1	0.7546	0.7912[1]	0.403
Low Ionic Strength_R2	1.1300	0.7985[1]	0.503
High Ionic Strength_R1	1.0241	0.8012[1]	0.476
High Ionic Strength_R2	0.9779	0.7974[1]	0.463
SRNOM2_R1	0.9083	0.7946[1]	0.444
SRNOM1_R2	1.1345	0.7955[2]	0.528
ESFA_R1	1.1272	0.7991[2]	0.502
DRO_R1	0.9670	0.7920[1]	0.458
DRO_R2	1.0433	0.7977[1]	0.480
DRO + High Ionic Strength_R1	1.1250	0.7995[1]	0.502

*Superscript in CAC/Quartz column indicates the CAC preparation batch that was used

Table S4. General timing of composite effluent sample collection.

Sample	Time (min)	Sample	Time (min)
1	5	17	30
2	5	18	30
3	5	19	30
4	5	20	30
5	5	21	30
6	5	22*	950-1200
7	10	23	1440
8	10	24	1440
9	10	25	1440
10	10	26	1440
11	10	27	1440
12	10	28	1440
13	20	29	1440
14	20	30	1440
15	20	31	1440
16	20		

*Sample 22 was collected between 16 and 19 hours after sample 21 depending on column start times

Table S5. Optimized MS/MS transitions, QqQ operating parameters, method detection limits (MDL) and corresponding isotopically labeled standards (ILS) for each PFAA. MDLs are reported for both external and internal calibrations.

PFAA	MRM Transitions (*m/z*)		MS Voltages		MDL *External* (μM)	MDL *ILS* (μM)	ILS
	Precursor ion	Product ion(s)	Fragmentor	Collision Energy			
**PFOA**	413	368.9*, 169	92	0, 12	0.00037	0.00088	13C8-PFOA
**PFPeA**	263	218.9	56	0	0.0011	0.00090	13C5-PFHxA
**PFBS**	298.9	99, 79.8*	100	29, 45	0.00078	0.00061	13C5-PFHxA
**FBSA**	297.9	78	100	20	0.00062	0.00046	13C5-PFHxA
ILS Information					***Final Conc. in Samples (μM)***		
**13C8-PFOA**	421.1	376	92	0	0.056		
**13C5-PFHxA**	218	273,120.3	56	0,12	0.043		

*Indicates quantifier ion if more than one product ion listed

Table S6. Freundlich adsorption isotherm parameters collected following protocols described in previous work.[1,4]

PFAS	Matrix	$\log K_F$ [μmol^{1-n}L^{n}g^{-1}]	n	$\log K_d$ [L/g] $(C_{aq} = 0.05\ \mu M)$	n	R^2
**PFOA**	1 mM $NaHCO_3$, pH = 5	2.28 (±0.04)	0.16 (±0.03)	3.43 (±0.21)	7	0.63
	1 mM $NaHCO_3$, pH = 7.5	2.26 (0.03)	0.19 (0.01)	3.32 (0.05)	10	0.99
**PFPeA**	1 mM $NaHCO_3$, pH = 5	1.70 (±0.05)	0.42 (±0.04)	2.46 (±0.11)	8	0.98
	1 mM $NaHCO_3$, pH = 7.5	1.70 (0.10)	0.54 (0.07)	2.30 (0.19)	10	0.97
**PFBS**	1 mM $NaHCO_3$, pH = 5	2.02 (±0.02)	0.26 (±0.02)	2.98 (±0.04)	8	0.99
	1 mM $NaHCO_3$, pH = 7.5	1.97 (0.06)	0.32 (0.04)	2.86 (0.11)	10	0.97
**FBSA**	1 mM $NaHCO_3$, pH = 5	2.13 (±0.04)	0.44 (±0.03)	2.86 (±0.07)	10	0.99
	1 mM $NaHCO_3$, pH = 7.5	1.81 (±0.04)	0.42 (±0.03)	2.57 (±0.08)	10	0.99

Table S7. Mass fraction of PFAS released in column effluents after 1,600 pore volumes for each influent condition based on the initial amount of PFAS pre-adsorbed to CAC**.** Control (1 mM $NaHCO_3$, pH = 7.5), high IS (100 mM, NaCl), low IS (10 mM, NaCl), SRNOM (10 mg/L, small MW), ESFA (10 mg/L, large MW), DRO (1,400 µg/L), and DRO with high IS (DRO = 1,400 µg/L, IS = 100 mM, NaCl). Standard deviation between duplicate experiments was calculated when available and asterisks represent results that were statistically significant from the control ($p < 0.05$).

Experiment	**PFOA**	**FBSA**	**PFBS**	**PFPeA**
Control	0.02 ± 0.01	0.30 ± 0.04	0.46 ± 0.04	0.83 ± 0.02
High IS	0.0089 ± 0.00	0.44 ± 0.07	0.77 ± 0.01*	0.89 ± 0.05
Low IS	0.0064 ± 0.00	0.36 ± 0.02	0.65 ± 0.09	0.74 ± 0.06
Low MW DOM	0.23 ± 0.01*	0.41 ± 0.02	0.69 ± 0.13	0.89 ± 0.04
High MW DOM	0.013	0.33	0.72	0.86
DRO	0.67 ± 0.04*	0.36 ± 0.05	0.71 ± 0.17	0.82 ± 0.12
DRO + High IS	0.69	0.39	0.83	0.89

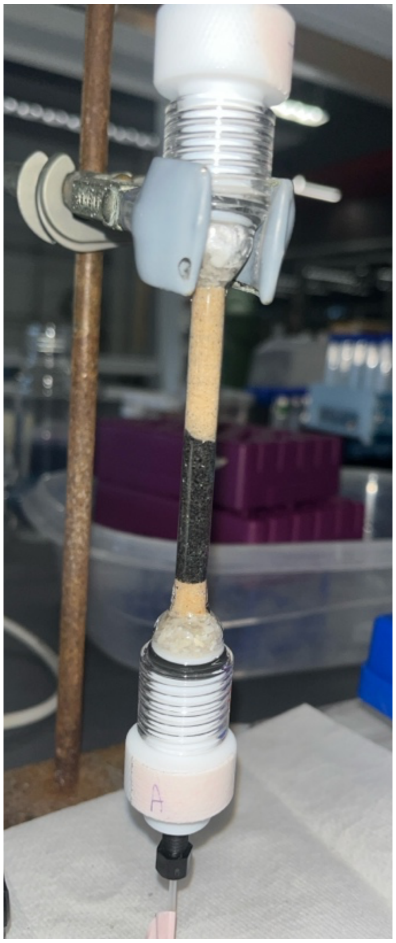

Figure S1. Column packed with clean quartz sand and 1% CAC/sand mixture with coarse Unimin sand at the inlet and outlet and glass wool at the outlet.

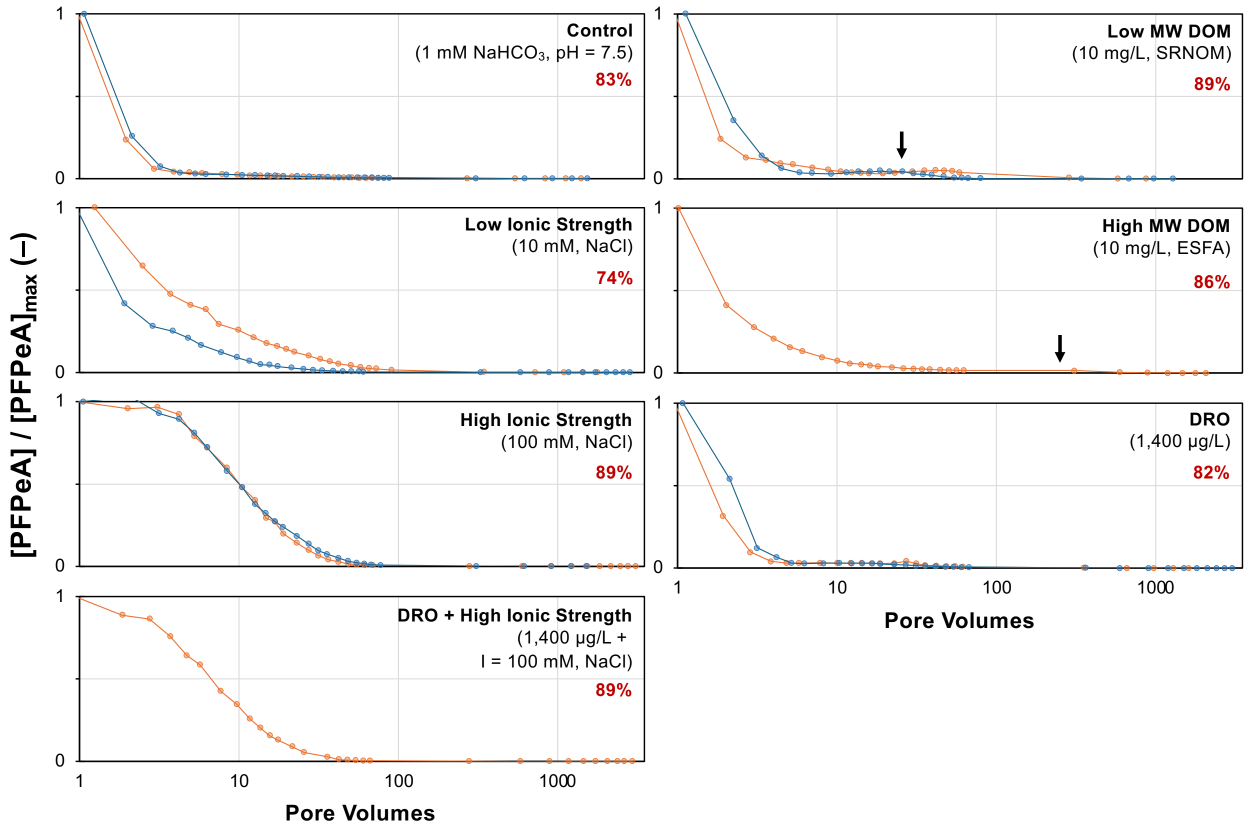

Figure S2. PFPeA full maximum value normalized effluent concentration profiles. Influent conditions are indicated in panel and the percent of total mass released for each condition is indicated in red below. Black arrows highlight the changing impact of DOM with different molecular weights. Blue and orange data points in each panel represent replicate experiments.

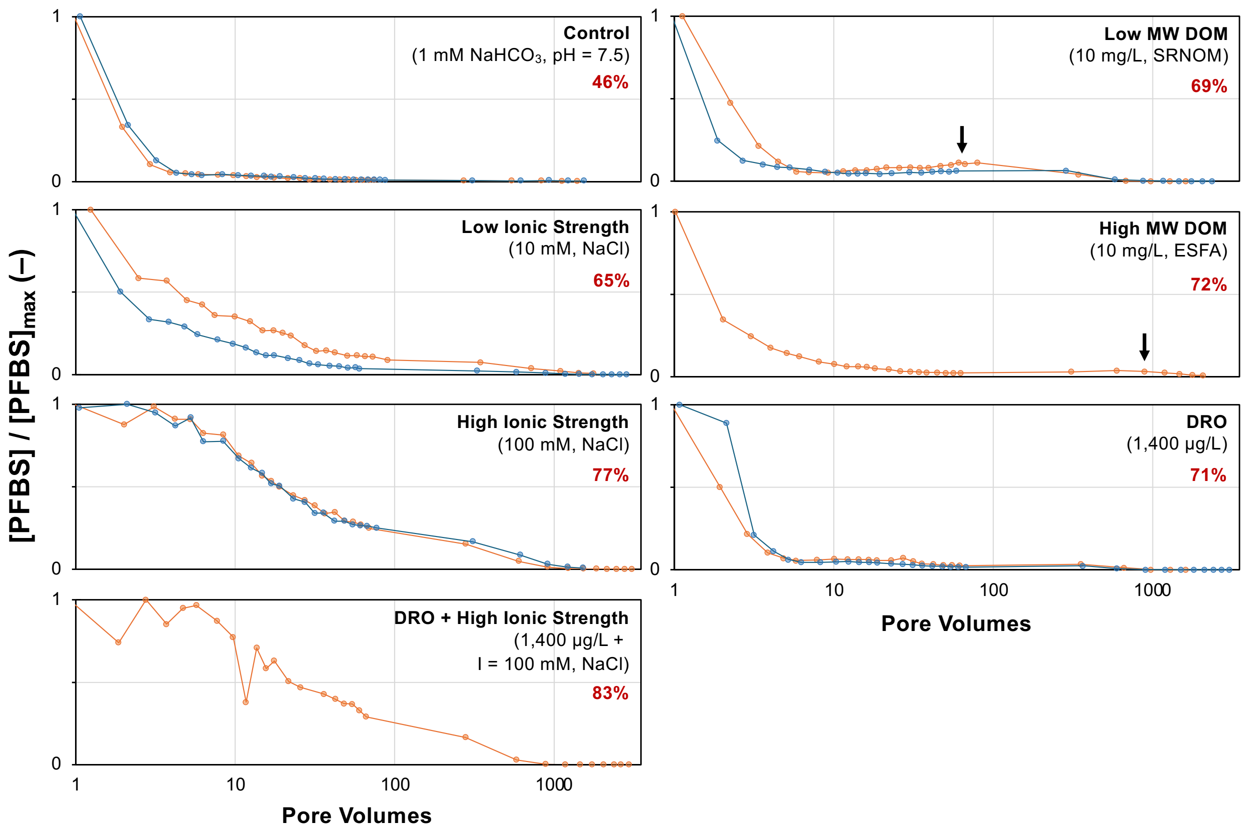

Figure S3. PFBS full maximum value normalized effluent concentration profiles. Influent conditions are indicated in panel and the percent of total mass released for each condition is indicated in red below. Black arrows highlight the changing impact of DOM with different molecular weights. Blue and orange data points in each panel represent replicate experiments.

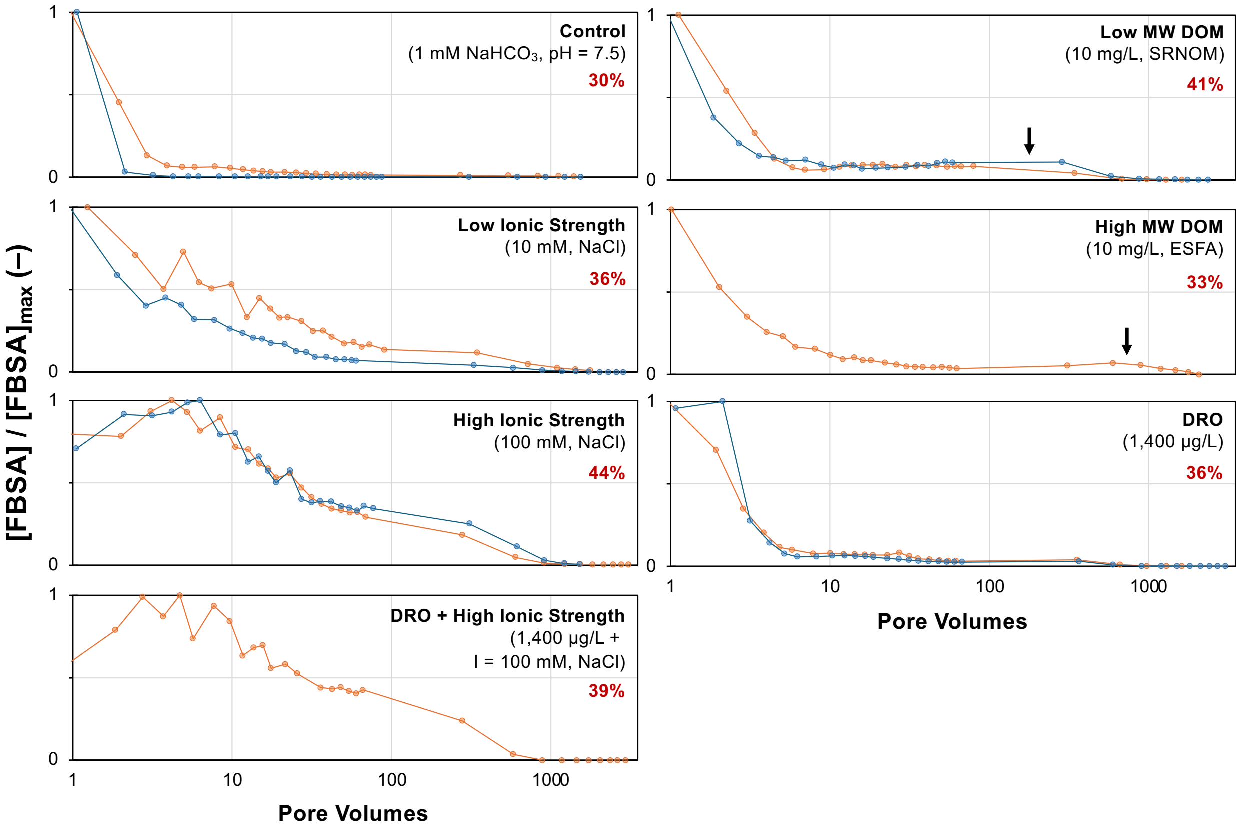

Figure S4. FBSA full maximum value normalized effluent concentration profiles. Influent conditions are indicated in panel and the percent of total mass released for each condition is indicated in red below. Black arrows highlight the changing impact of DOM with different molecular weights. Blue and orange data points in each panel represent replicate experiments.

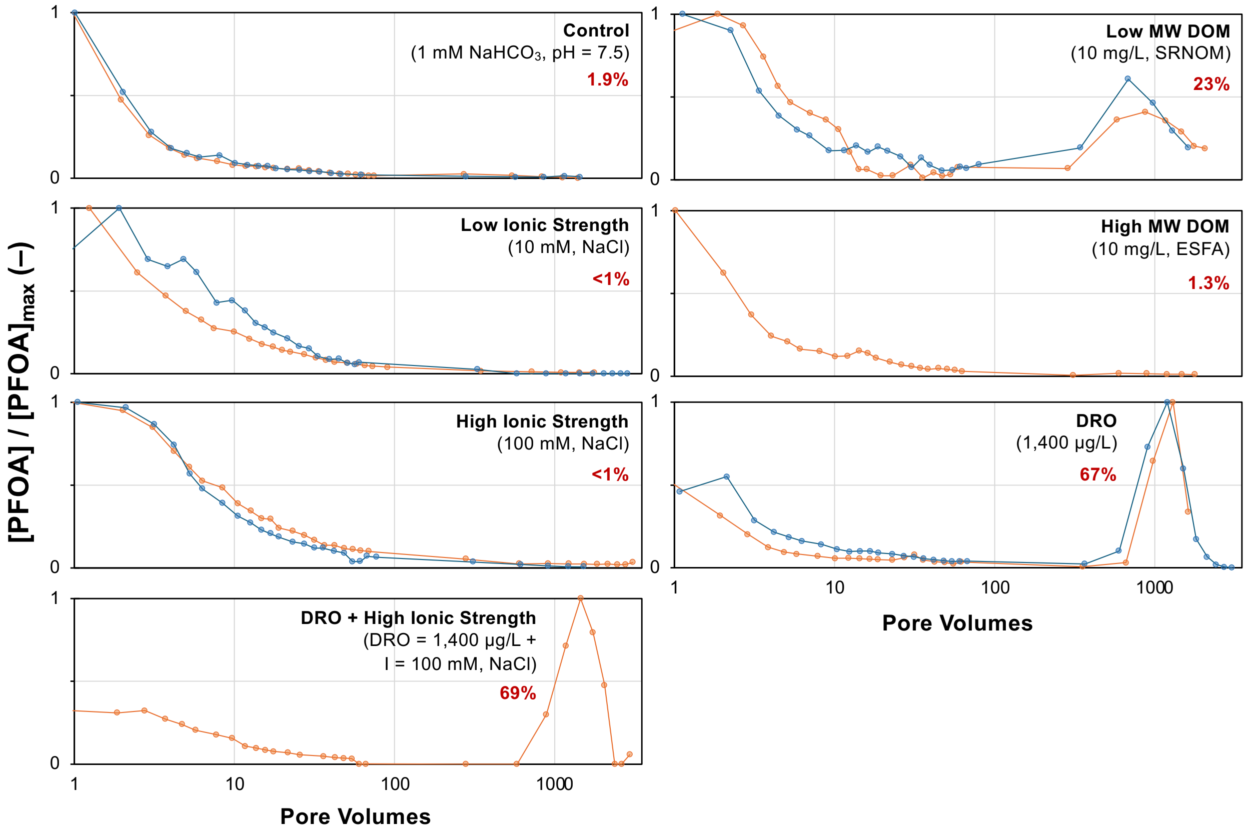

Figure S5. PFOA full maximum value normalized effluent concentration profiles. Influent conditions are indicated in panel and the percent of total mass released for each condition is indicated in red below. Blue and orange data points in each panel represent replicate experiments.

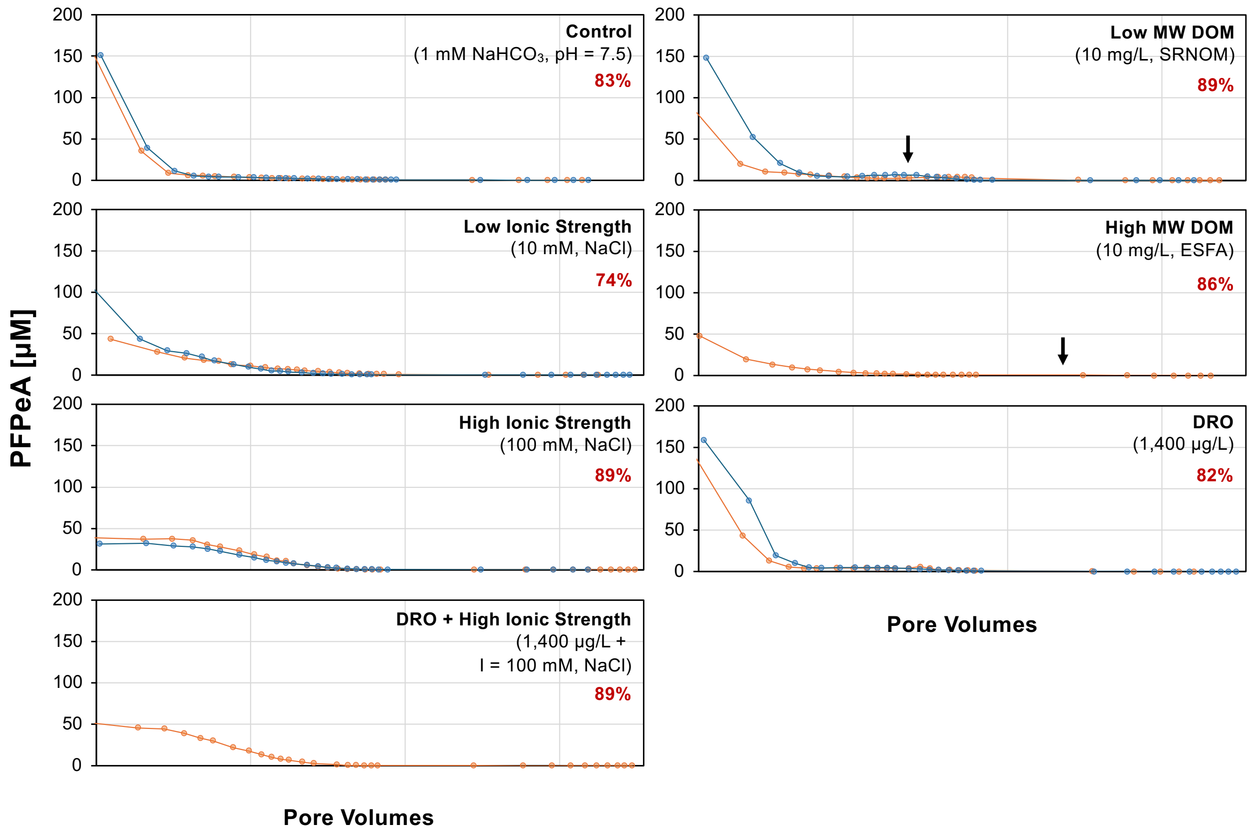

Figure S6. PFPeA full maximum value raw effluent concentration profiles. Influent conditions are indicated in panel and the percent of total mass released for each condition is indicated in red below. Black arrows highlight the changing impact of DOM with different molecular weights. Blue and orange data points in each panel represent replicate experiments.

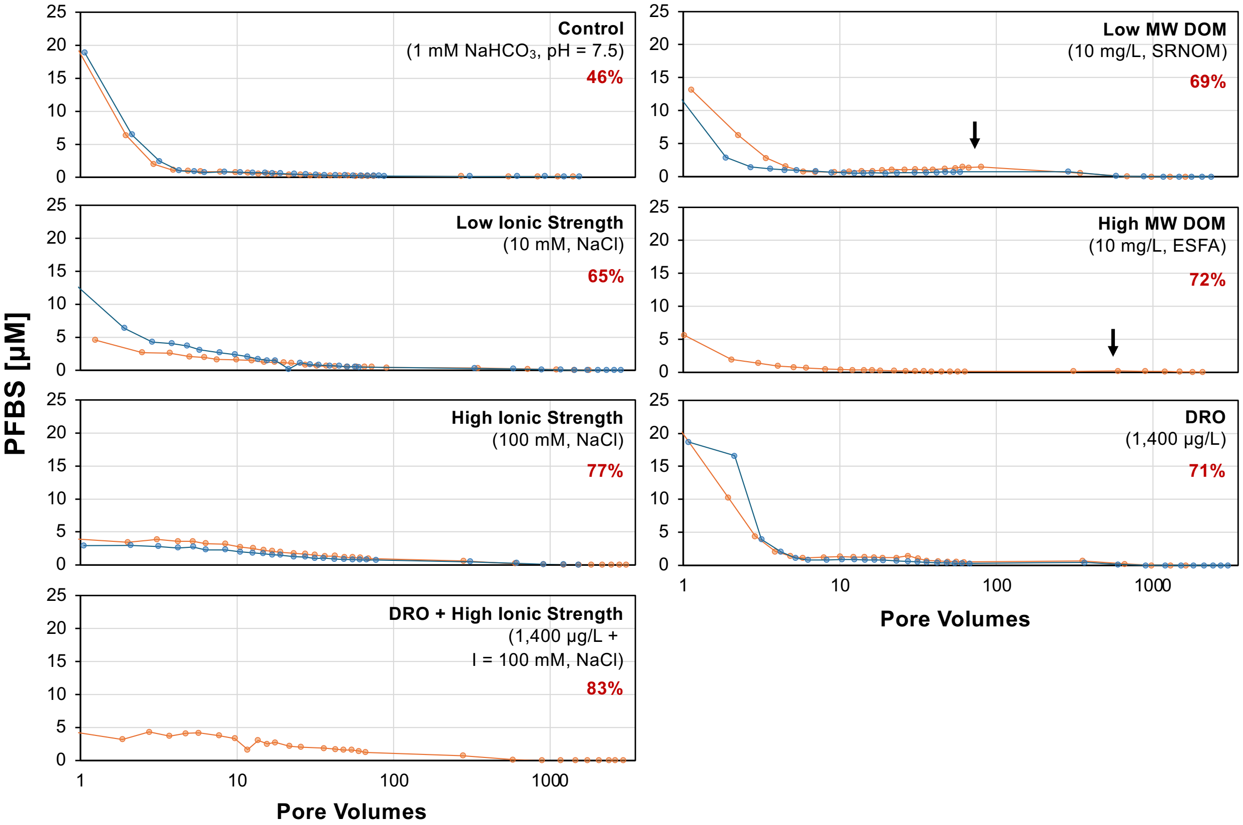

Figure S7. PFBS full maximum value raw effluent concentration profiles. Influent conditions are indicated in panel and the percent of total mass released for each condition is indicated in red below. Black arrows highlight the changing impact of DOM with different molecular weights. Blue and orange data points in each panel represent replicate experiments.

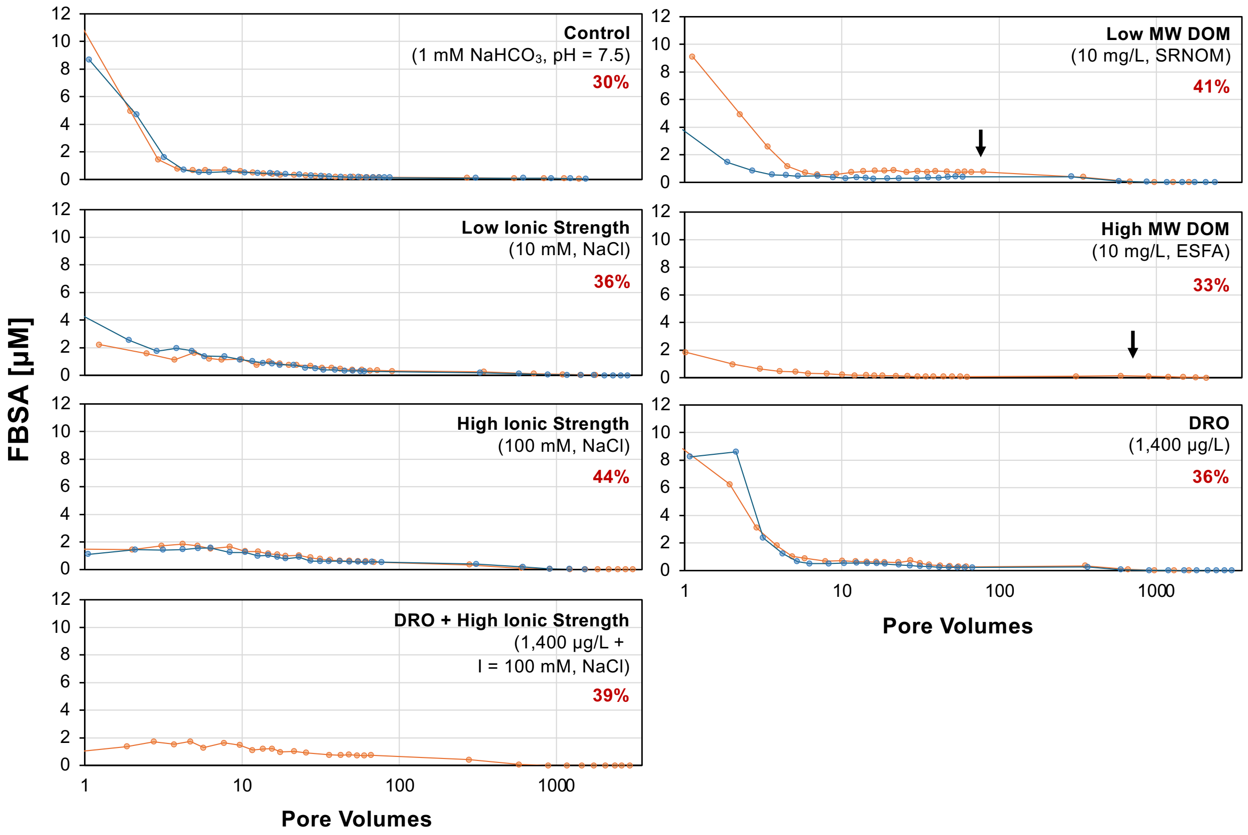

Figure S8. FBSA full maximum value raw effluent concentration profiles. Influent conditions are indicated in panel and the percent of total mass released for each condition is indicated in red below. Black arrows highlight the changing impact of DOM with different molecular weights. Blue and orange data points in each panel represent replicate experiments.

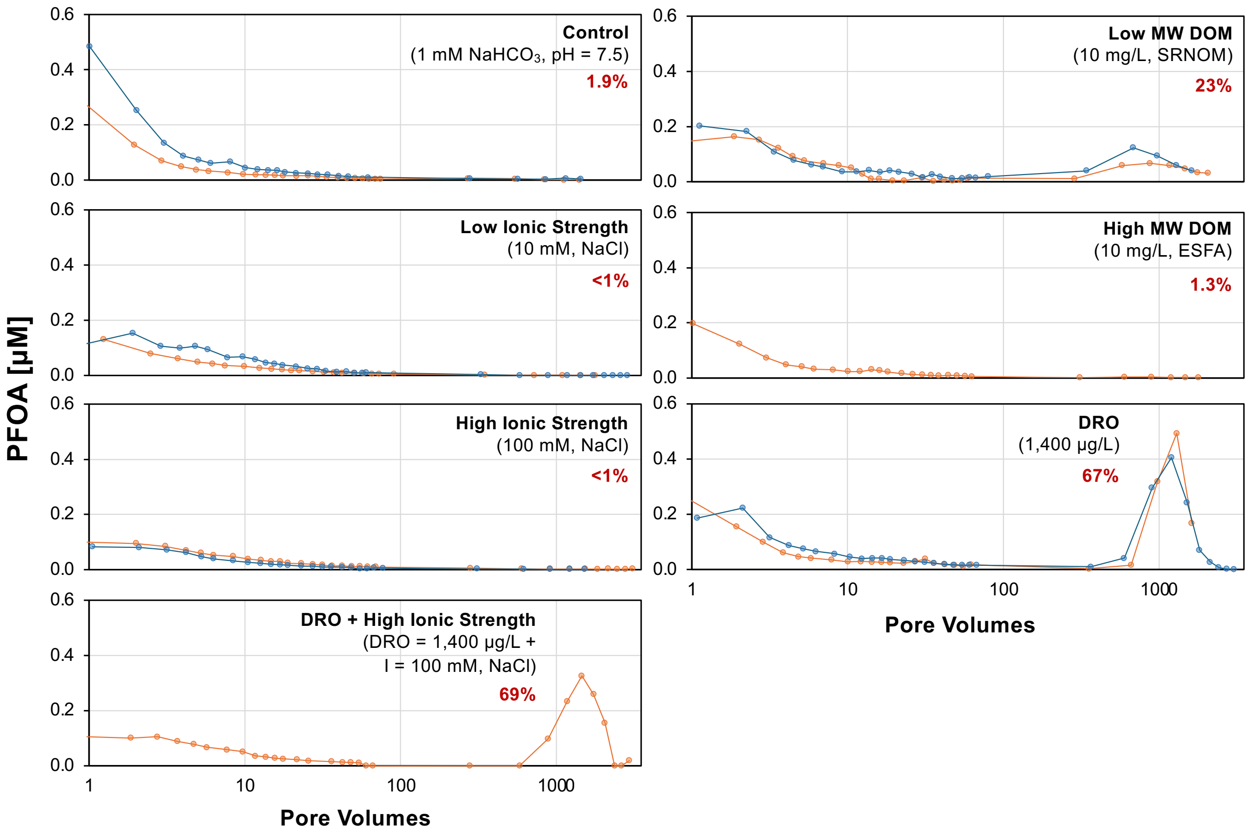

Figure S9. PFOA full maximum value raw effluent concentration profiles. Influent conditions are indicated in panel and the percent of total mass released for each condition is indicated in red below. Blue and orange data points in each panel represent replicate experiments.

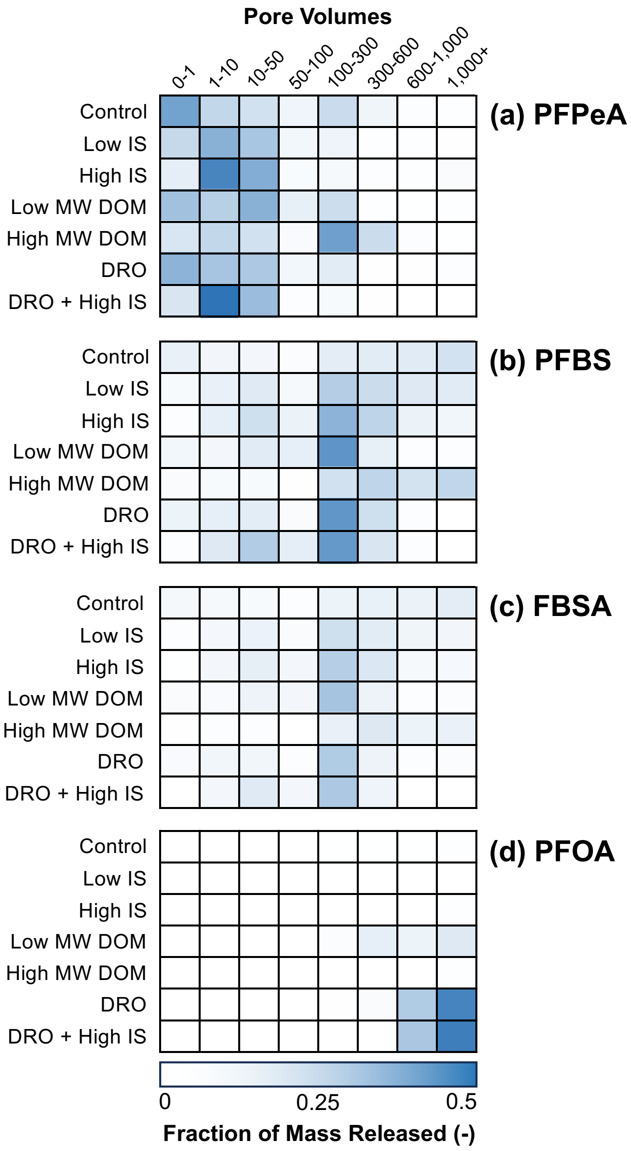

Figure S10. Fraction of total PFAS mass recovered in 0-1, 1-10, 10-50, 50-100, 100-300, 300-600, 600-1,000, and 1,000+ pore volume bins for each influent chemistry condition. The average mass fraction in the pore volume bin was calculated when replicates were performed.

References

(1) Molé, R. A.; Velosa, A. C.; Carey, G. R.; Liu, X.; Li, G.; Fan, D.; Danko, A.; Lowry, G. V. Groundwater Solutes Influence the Adsorption of Short-Chain Perfluoroalkyl Acids (PFAA) to Colloidal Activated Carbon and Impact Performance for in Situ Groundwater Remediation. *J. Hazard. Mater.* **2024**, *474*, 134746. https://doi.org/10.1016/j.jhazmat.2024.134746.
(2) Louie, S. M.; Spielman-Sun, E. R.; Small, M. J.; Tilton, R. D.; Lowry, G. V. Correlation of the Physicochemical Properties of Natural Organic Matter Samples from Different Sources to Their Effects on Gold Nanoparticle Aggregation in Monovalent Electrolyte. *Environ. Sci. Technol.* **2015**, *49* (4), 2188–2198. https://doi.org/10.1021/es505003d.
(3) Pavlik, J. W.; Perdue, E. M. Number-Average Molecular Weights of Natural Organic Matter, Hydrophobic Acids, and Transphilic Acids from the Suwannee River, Georgia, as Determined Using Vapor Pressure Osmometry. *Environ. Eng. Sci.* **2015**, *32* (1), 23–30. https://doi.org/10.1089/ees.2014.0269.
(4) Molé, R. A.; Correia de Velosa, A.; Liu, X.; Li, G.; Fan, D.; Danko, A.; Lowry, G. V. Impacts of Groundwater Constituents and Colloidal Activated Carbon (CAC) Surface Chemistry on the Adsorption of Perfluoroalkyl Acids (PFAA) in Aqueous Film-Forming Foam (AFFF)-Impacted Groundwater. *ACS EST Water* **2025**. https://doi.org/10.1021/acsestwater.5c00236.
